# Biomimetic Tissue Engineering Strategies for Craniofacial Applications

**DOI:** 10.3390/biomimetics9100636

**Published:** 2024-10-18

**Authors:** Isis Fatima Balderrama, Sogand Schafer, Muhammad El Shatanofy, Edmara T. P. Bergamo, Nicholas A. Mirsky, Vasudev Vivekanand Nayak, Elcio Marcantonio Junior, Adham M. Alifarag, Paulo G. Coelho, Lukasz Witek

**Affiliations:** 1 Department of Diagnosis and Surgery, School of Dentistry of Araraquara, Sao Paulo State University, Sao Paulo 14801-385, Brazil; 2 Biomaterials Division, NYU Dentistry, New York, NY 10010, USA; 3 Division of Plastic, Reconstructive and Oral Surgery, Children’s Hospital of Philadelphia, Philadelphia, PA 19104, USA; 4 Department of Otolaryngology, University of Miami Miller School of Medicine, Miami, FL 33136, USA; 5 Department of Prosthodontics, NYU Dentistry, New York, NY 10010, USA; 6 University of Miami Miller School of Medicine, Miami, FL 33136, USA; 7 Department of Biochemistry and Molecular Biology, University of Miami Miller School of Medicine, Miami, FL 33136, USA; 8 Department of General Surgery, Temple University Hospital System, Philadelphia, PA 19140, USA; 9 Division of Plastic Surgery, DeWitt Daughtry Family Department of Surgery, University of Miami Miller School of Medicine, Miami, FL 33136, USA; 10 Department of Biomedical Engineering, NYU Tandon School of Engineering, Brooklyn, NY 11201, USA; 11 Hansjörg Wyss Department of Plastic Surgery, NYU Grossman School of Medicine, New York, NY 10016, USA

**Keywords:** biomimetics, tissue engineering, regenerative medicine, biomaterials

## Abstract

Biomimetics is the science of imitating nature’s designs and processes to create innovative solutions for various fields, including dentistry and craniofacial reconstruction. In these areas, biomimetics involves drawing inspiration from living organisms/systems to develop new materials, techniques, and devices that closely resemble natural tissue structures and enhance functionality. This field has successfully demonstrated its potential to revolutionize craniofacial procedures, significantly improving patient outcomes. In dentistry, biomimetics offers exciting possibilities for the advancement of new dental materials, restorative techniques, and regenerative potential. By analyzing the structure/composition of natural teeth and the surrounding tissues, researchers have developed restorative materials that mimic the properties of teeth, as well as regenerative techniques that might assist in repairing enamel, dentin, pulp, cementum, periodontal ligament, and bone. In craniofacial reconstruction, biomimetics plays a vital role in developing innovative solutions for facial trauma, congenital defects, and various conditions affecting the maxillofacial region. By studying the intricate composition and mechanical properties of the skull and facial bones, clinicians and engineers have been able to replicate natural structures leveraging computer-aided design and manufacturing (CAD/CAM) and 3D printing. This has allowed for the creation of patient-specific scaffolds, implants, and prostheses that accurately fit a patient’s anatomy. This review highlights the current evidence on the application of biomimetics in the fields of dentistry and craniofacial reconstruction.

## 1. Introduction

Biomimetics originates from the words “bio”, meaning “life”, and “mimesis”, meaning “imitation” [1,2]. The first evidence of biomimetics originated in the Roman and pre-Colombian cultures of South America, where crude dental implants were used for the first time [3]. The applicability of biomimetics has come a long way since and is an active area of research among dentists and craniofacial reconstructive surgeons. The objective of biomimetic restorative dentistry is to synthesize materials that have the potential to replicate and restore components of teeth such as enamel, dentin, cementum, periodontal ligament, and bone [2,4]. This is in contrast to the conventional approach, in which the affected and surrounding tooth structures are removed for subsequent replacement [3]. Such treatment modalities generally shorten the lifespan of the surrounding tooth structures. Biomimetics, on the other hand, is a conservative approach aimed at restoring teeth and stimulating natural tissues. Using a combination of stress-reducing and bond-maximizing protocols, a biomimetic approach aims to carefully repair damaged or missing tissue while retaining the structures’ inherent natural properties [3].

Although biomimetics was originally developed within the field of dentistry, it has now been extended to the repair of craniofacial defects [5]. Craniofacial reconstruction is centered around four primary goals: to restore function, form, esthetics and to permit craniofacial growth (especially in the case of pediatric patients). Currently, the gold standard for craniofacial reconstruction involves the utilization of autologous bone [3]. Advantages of this approach include the remarkable ability for osseointegration, resistance to infection, and the capability of permitting normal growth in the craniofacial skeleton. However, these surgeries are typically associated with donor site morbidity, especially when composite defects present a combination of missing skin, bone, and/or dura, and chimeric free flaps may be necessary [6]. Given these disadvantages, tissue engineering research has recently focused its attention on the area of craniofacial reconstruction. The objective of this study was to review the literature on advances in biomimetics in the fields of dentistry and craniofacial reconstruction.

## 2. Biomimetics in Dentistry

The importance of regenerating the damaged or missing/lost oral tissue with a replacement through materials not only ensures a better prognosis, but also improves biocompatibility and, thereby, the overall success rate of the treatment. The goal of the biomimetic concept is to develop restorative materials that correlate with the natural mechanisms of the oral environment that mimic, preserve, or restore the biomechanics of the natural tissue structures [2]. As such, biomimetic aspects in dentistry and craniofacial surgery are multifactorial and can be applied to various sub-specialties in the field (summarized in Table 1).

### 2.1. Restorative Dentistry and Prosthodontics

The biomimetic mineralization of enamel and dentin is a current approach in restorative dentistry and is considered to involve an enamel-like fluorapatite layer on a mineral substrate that has the potential to enable the remineralization of superficial enamel defects on the exposed dentin [7,8]. The objective is to produce synthetic materials that mimic the dentinal tissue’s microstructure and mechanical performance for tissue preservation and adhesion [9,10]. Biomimetic remineralization of dentin can be performed using ion-containing solutions or ion-leaching silicon, calcium–silicate hybrids, or agarose gels. These methods provide the experimental basis for dentin’s remineralization and the treatment of dentin hypersensitivity and dental caries [11,12]. Glass ionomer cements are also considered biomimetic materials due to their dentin-like properties, capacity to adhere to tooth structures, and ability to release fluoride [13].

On the other hand, resin composites have witnessed significant improvements in recent years, making them a popular choice in restorative dentistry [14,15,16,17]. These improvements primarily focus on enhancing their physical and mechanical properties, ensuring their long-term durability and esthetic outcomes [14,15,16,17]. One notable advancement in resin composites is in the development of nanofillers, such as nanoparticles and nanoclusters, which have greatly enhanced the material’s mechanical properties [14,15]. These fillers offer higher strength, wear resistance, and elasticity compared with traditional fillers, leading to the enhanced functional longevity of dental restorations. Moreover, nanofillers also contribute to improved esthetics by achieving a more natural appearance, making them suitable for a wide range of clinical scenarios [14,15].

In addition to physical and mechanical improvements, advancements in resin composites have also focused on biocompatibility and adhesion properties. The development of adhesive systems has revolutionized modern restorative dentistry, enabling the bonding of restorative materials to tooth structures. These adhesive systems provide a reliable bond and prevent microleakage, leading to better restoration longevity [18,19]. Enhancing biocompatibility ensures that resin composites have minimal adverse effects on the surrounding oral tissues, promoting patient comfort and health. Bioactive resin composites and adhesives have garnered significant attention in the field of dental materials [20,21,22,23]. They consist of a resin matrix and bioactive fillers such as glass or ceramic particles. The bioactive properties of these materials are derived from their ability to release calcium, phosphate, and fluoride ions when exposed to saliva or an aqueous environment, thereby mimicking the natural remineralization process of the tooth structure. Furthermore, these materials facilitate the inhibition of bacterial growth and the remineralization of damaged tooth structures, reducing the risk of recurrent caries [20,21].

Advances in technology and materials science have significantly enhanced indirect restorative systems and oral rehabilitation treatments, offering more reliable workflows that result in successful esthetic and functional outcomes. Ceramics have become a cornerstone in various areas of dentistry, particularly in dental implants and prostheses, due to their biocompatibility, esthetic appeal, and chemical stability. Additionally, their mechanical properties, such as strength, fracture resistance, and wear resistance, make ceramic systems highly suitable for widespread clinical use [24]. The balance between translucency, strength, and wear resistance also contributes to their intraoral durability [25,26,27,28,29]. Metal ceramics, first introduced in the 1960s, were the initial indirect restorative option to be widely adopted in clinical practice for restoring severely damaged teeth. The brittleness and low strength of early ceramic materials necessitated the use of metal frameworks to improve overall strength and toughness, which expanded their clinical applications [24]. The literature extensively supports the use of metal ceramics, citing excellent survival rates in various clinical situations [26,28,30,31]. Metal ceramics remain the preferred choice for long-span partial and full-arch fixed dental prostheses (FDPs), especially in cases involving high occlusal forces, parafunctional habits, or unfavorable biomechanical support [25]. Previous reports present high survival rates of 94–98% for both tooth- and implant-supported metal ceramic restorations after an observation time of 5 years [26,28,30,31]. However, some complications, such as secondary caries, tissue inflammation, ceramic fractures, and debonding, have been reported [26,28,30,31].

**Figure 1:**
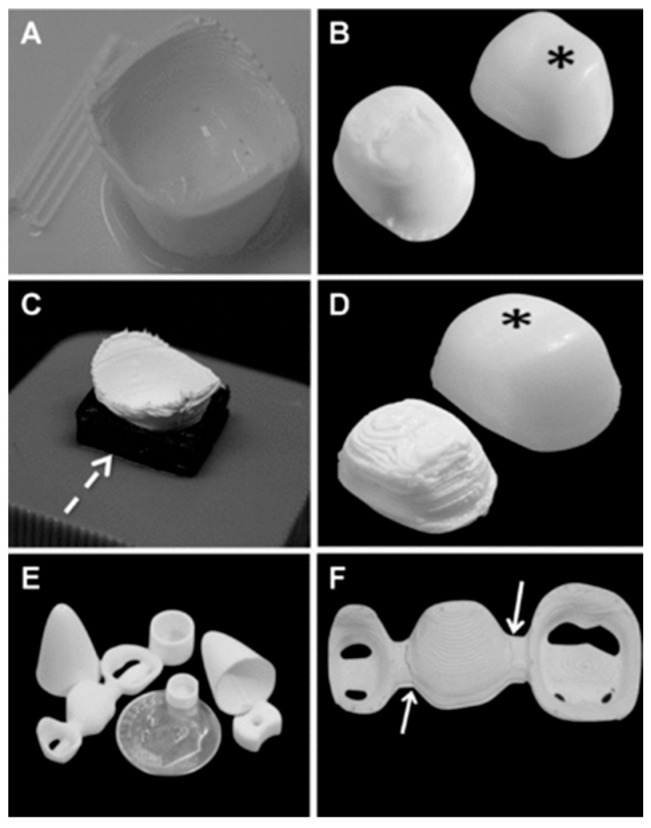
Three dimensional (3D)-printed zirconia. (**A**) A printed core comprised of zirconia without support material, synthesized through the inversion of the stereolithography (.stl) file used to design the construct. (**B**) Flattened top-surface post-sintering and side-by-side comparison to a commercially available core (indicated by the black asterisk). (**C**) The use of a fugitive (removable) support material (carbon black—shown by a segmented white arrow) to build a support for the crown. (**D**) Post-sintering pictograph of the printed core with a commercial core (indicated with black asterisk) as an example for comparison. (**E**) Pictograph of various robocasted (3D-printed) specimens and (**F**) a three-unit FDP zirconia framework post-sintering, with crack lines shown in white arrows. Reprinted with permission from the work by Silva et al. [51]. Copyright 2010 by The American College of Prosthodontists. Published by John Wiley & Sons Ltd.

All-ceramic materials are becoming more prevalent due to the wider use of different processing modalities, such as CAD/CAM manufacturing, 3D-printing manufacturing (Figure 1), and hybrid manufacturing using digital workflows [32]. Dental ceramics are used for indirect restoration, focusing on the prosthodontics field, especially for the fabrication of tooth- and implant-supported crowns and fixed partial dentures [26,27,28,29,30,31]. All ceramic systems have been classified as (i) glass-matrix ceramics (which include feldspathic ceramics, synthetic ceramics [lithium disilicate (Li_2_O_5_Si_2_) systems], and glass-infiltrated ceramics); (ii) polycrystalline ceramics (which include alumina, zirconia, and alumina–zirconia composites); and (iii) resin-matrix ceramics (which include polymer matrices containing inorganic ceramic compounds such as feldspathic ceramic and glass ceramics) [33]. Glass ceramics are known for their high esthetic results, mimicking the color, opalescence, and translucency of natural teeth [34,35,36]. Each type has different mechanical properties and applications, with lithium disilicate systems being the most commonly used due to their balance between strength and esthetics [37,38,39]. Glass ceramics present a similar elastic modulus relative to dental enamel and are widely used for laminate veneers and monolithic crowns with favorable biomechanical performance and high success rates. Laminate veneers have shown a survival rate of 97.4% after 10 years, with a low complication rate of ~1.65% [40]. Similarly, crowns with minimally invasive or conventional preparations have presented a survival rate of 95–100% in the medium term (5–8 years), with minor reparable chipping being the most common complication [29,31,41].

Polycrystalline ceramics, particularly zirconia systems, have revolutionized dental restorations due to their favorable mechanical properties and biocompatibility. These materials, when used in dental prosthesis fabrication, offer high strength/toughness, which makes them suitable for wide range of applications in oral rehabilitation. Despite the excellent mechanical properties of zirconia systems, technical complications such as veneering ceramic fractures have been a common issue reported in clinical studies [25,26,28,30,31,42]. This has led to the development of translucent zirconia systems to improve esthetics while maintaining adequate mechanical properties, allowing for the fabrication of full-contour restorations without a veneering ceramic [43]. Additionally, multilayered zirconia systems have been proposed to mimic the natural gradient of the tooth structure, providing a balance of translucency and strength in full-contour restorations [44,45]. Regarding monolithic, full-contour zirconia restorations, recent systematic reviews have demonstrated that both tooth-/implant-supported single crowns and fixed partial dentures show excellent medium-term survival rates, ranging from 96–100%, and 99.6%, respectively, over a follow-up period of 3–5 years. Minor chipping has been identified as the most common complication [46,47]. Furthermore, monolithic zirconia has emerged as a viable alternative to traditional metal frameworks in full-arch prostheses, with studies reporting 100% survival and success rates after up to two years of follow-up [48,49]. However, additional research is needed to fully validate the broader clinical application of these systems. Advances in polymer matrices have also reinvigorated restorative systems by facilitating the use of pre-processed discs or blocks for CAD/CAM fabrication and contributing to the development of 3D-printed materials [50].

Innovations in material composition with a high (>60% by weight) inorganic content, which results in increased flexural strength, have broadened their clinical applications [52]. In addition, the biomechanical behavior of resin-matrix ceramics provides favorable resilience and improves damage tolerance [53,54]. However, clinical studies are important to evaluate the long-term performance of resin-matrix-based restorations. Improvements in the indirect restorative systems have revolutionized the field of dentistry, offering patients a durable, esthetic, and long-lasting solution for restoring teeth. These advancements have not only improved the quality of dental care but have also enhanced the overall patient experience. As technology continues to evolve, exciting developments in indirect restorative systems for dentistry, such as the development of bioactive indirect restorative systems and different types of multilayered materials mimicking natural teeth properties are expected.

### 2.2. Endodontics

**Figure 2:**
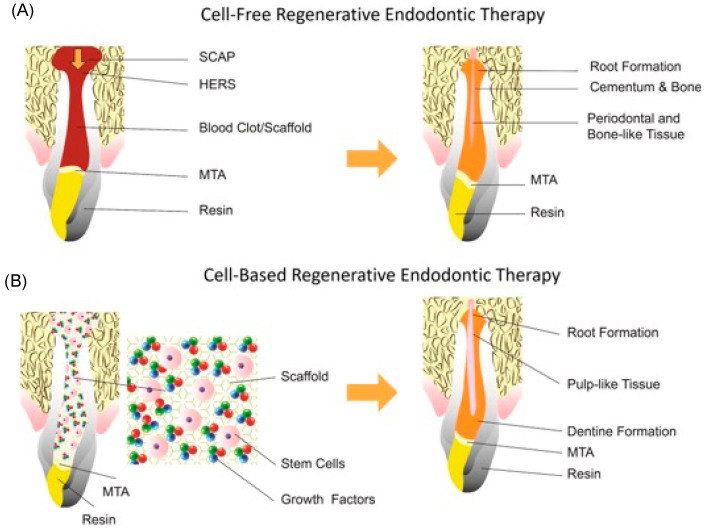
Schematic illustration of (**A**) cell-free, and (**B**) cell-based endodontic therapy. Cell-based endodontic therapy includes the use of stem cells, scaffolds, and growth factors. Abbreviations: MTA: mineral trioxide aggregate; HERS: Hertwig’s epithelial root sheath; SCAP: stem cells from the apical papilla. Reprinted with permission from the work by Lin et al. [56]. Copyright 2021 International Endodontic Journal. Published by John Wiley & Sons Ltd.

Dental trauma or bacterial contamination in/of the pulp tissue of teeth can increase pulp tissue inflammation and, subsequently, pulp necrosis [55]. The field of regenerative endodontics (Figure 2) involves studying the structure and function of natural teeth to design materials and techniques that closely mimic native properties, with more effective and durable treatments for patients with dental necrosis.

One area in which biomimetics has had a significant impact in endodontics is in the development of bioactive materials for root canal therapy. Traditional root canal treatments involve removing the infected or damaged dental pulp and filling the root canal with an inert material to prevent reinfection [57]. However, these materials do not interact with the surrounding tissues or promote healing. Biomimetic materials, on the other hand, are designed to stimulate the natural healing processes in the dental pulp and to promote the regeneration of healthy tissues [58]. Cements have been developed with remineralization properties; however, one example of a biomimetic cement used in endodontics is with the incorporation of bioactive glasses [59,60]. Bioactive glass has the capacity to bond with living tissues and to promote mineralization, similar to the process of natural tooth formation. When placed in a root canal, bioactive glass can stimulate the growth of new dental tissues and help repair damage caused by infection or trauma. This can lead to better outcomes for patients and reduce the need for additional treatments in the future [59,60].

The synthesis of novel extracellular matrix (ECM)-mimicking structures with adequate chemical properties and porous, three-dimensional architectures has also been proposed in endodontics therapy to provide mechanical support and to regulate cell functions [61]. A biomimetic microenvironment of pulp–dentin has been described as a key concept in tissue engineering based on regenerative endodontics. These environments, composed of a synthetic nanoscale polymeric fiber structure, mimic pulp by providing a natural ECM and function as scaffolds of the pulp–dentin tissue complexes [62]. Calcium hydroxide is frequently used for several endodontic applications, including pulp-capping approaches and bactericidal effects conferred by its pH [63,64].

Dental pulp tissue engineering has demonstrated the ability of dental pulp stem cells to improve the differentiation into odontoblasts and to regenerate the dental pulp, which are useful approaches for necrotic teeth [65]. Stem-cell-based regenerative therapies using cells from human exfoliated deciduous teeth (SHED) into scaffolds for transplantation have resulted in the generation of tissue with similar morphological properties to those observed in human dental pulp [66]. Another biomimetic application is the use of nanofibrous scaffolds for regenerative endodontics, which can be applied in the intracanal drug delivery of antibiotics or in applications promoting dentin–pulp regeneration. Intracanal drug delivery can be applied in the context of disinfection, using antibiotic-containing polymer nanofibers and their microbial properties [67]. Electrospun polymer nanofibers have been applied in this technique, providing an antibiotic concentration and slow drug release to eradicate infection and to create a bacteria-free environment for tissue regeneration processes [68,69]. These nanofiber membranes can build a pro-regenerative biomimetic extracellular matrix and promote dentin–pulp regeneration while simultaneously encompassing the five tissue requirements, including pulp connective-tissue formation, dentin formation, revascularization, reinnervation, and radicular edification [70,71]. Inserting biodegradable three-dimensional scaffolds to carry stem cells with growth factors has been indicated as one of the most suitable approaches for dental pulp regeneration [72].

### 2.3. Periodontics and Implant Dentistry

Periodontal disease is one of the advanced diseases that causes tooth loss in adults. It is a chronic multifactorial inflammatory disease associated with dysbiosis plaque biofilms that destroy soft and hard tissue [73,74]. Strategies of periodontal therapy have included eliminating inflamed tissues caused by bacterial plaque through scaling and root planning (SRP) or open flap debridement and regenerating new periodontal tissue [75]. In periodontics, tissue engineering principles are employed to regenerate lost tissue and recover the functionality of damaged/lost tissues and organs. This approach involves the integration of three key components: scaffolds, cells, and cell signaling molecules, all of which work together to promote tissue repair and regeneration [74,76]. Matrix and growth factors, such as enamel-matrix derivates (Straumann^®^ Emdogain^®^; Straumann, Basel, Switzerland) and brain-derived neurotrophic factor, have been shown to modulate the rate of cementum and periodontal ligament tissue formation, contributing to accelerated regeneration of periodontal tissue [77]. Several other growth factors have now been developed for periodontal tissue regeneration, but their effectiveness still needs to be addressed.

The tissue engineering approach in periodontal regeneration uses carriers and/or membranes, known as guided-bone regeneration (GBR), to avoid epithelial tissue migration during healing and/or to release growth factors around the healing site. Biomimetic multifunctional growth-enhancing regenerative membranes demonstrate that a membrane with incorporated growth factors enhances bioactivity while also supporting osteoblast proliferation and differentiation [78]. The electrospinning technique has great potential for periodontal regeneration approaches, where various polymers are generated in nano-layers to produce scaffolds with functionally similar ECM characteristics [79]. For example, Tang et al. successfully manufactured a polymer/hydroxyapatite electrospun scaffold integrated with amoxicillin for its antimicrobial properties, demonstrating no signs of epithelial cell infiltration on the defect side of the membrane [80]. While many investigators have shown the effectiveness of this technique in preclinical studies, there is a paucity of research undergoing human trials [81].

The alveolar bone, which depends on the presence of tooth roots for the maintenance of its volume and density, begins to resorb almost immediately after tooth extraction. Socket preservation techniques are therefore utilized to minimize this bone loss, thereby providing a sound foundation for subsequent prosthetic rehabilitation, such as dental implants. These techniques employ the implantation of materials, such as bone grafts, into the defect space. Bone grafts can be autogenous, allogenic, xenogeneic, or synthetic [1]. Biomaterial integration with cells and bioactive molecules have been proposed to improve clinical outcomes and to preserve the ridge anatomy. For example, a notable study demonstrated the integration of cells and bioactive molecules for enhanced alveolar preservation, where the combination of bone morphogenetic proteins with scaffold materials and novel cell lines optimized regenerative outcomes by promoting three-dimensional bone healing [82]. Regarding dental implants, the approach of biomimetics aims to attain a level of osseoconduction and osseoinduction capacity to mimic the natural structure of the tissue during treatment [83]. Commercially, titanium materials are commonly used for dental implants due to their biocompatibility, corrosion resistance, and mechanical properties; however, technological advances in ceramics demonstrate that zirconia implants also have a strong capacity for bone tissue integration [84,85].

**Figure 3:**
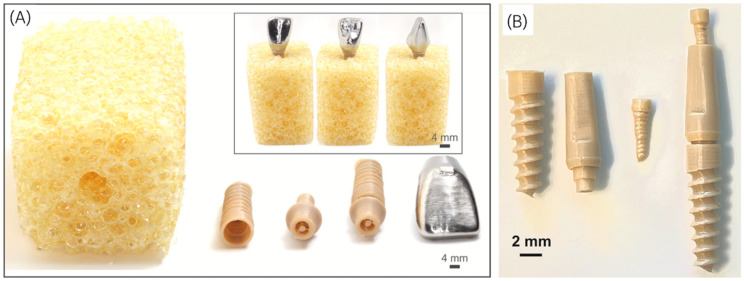
Pictographs of novel 3D-printed PEEK implants placed within a faux bone block (**A**) with abutments and (**B**) without abutments. Reprinted with permission from the work by Sonaye et al. [88]. Copyright 2022 Elsevier Ltd.

Another option for replacing metallic implants is a polymeric biomaterial called polyether ether ketone (PEEK) (Figure 3), which demonstrates favorable biocompatibility, chemical resistance, and similar mechanical properties relative to human bone [86,87,88]. The design of implant hardware and the surgical technique associated with it have had a significant impact on osseointegration healing pathways. This impact is seen not only in the modulation of primary stability levels and the peri-implant tissue response but also in the interaction of surface modifications with the surrounding host environment. Consequently, these factors affect the healing kinetics and biomechanical competence [89,90,91]. When it comes to the implant body design, a biomimetic conical design has been shown to provide a higher insertion torque compared with cylindrical implants. This finding is supported by histomorphometric data suggesting that this design leads to improved osseointegration [92]. Furthermore, the thread profile has been evolving over time, with the aim of optimizing the biomechanical performance of the implants, especially in compromised bone scenarios, favoring implant primary stability, stress distribution, and the healing response [93,94,95,96,97]. At a favorable thread pitch dimension, ranging from 0.8 to 1.6 mm, the axial load of implants with square and buttress threads is mostly dissipated through compressive stresses, while implants with V-shaped and reverse-buttress threads transmit the axial load through a combination of compressive, tensile, and shear stresses [94]. Thus, they are more favorable configurations for dental implants, especially when dealing with low-density-bone scenarios [93,98].

Another aspect of improving the biological response and healing process that has been extensively studied is the relationship between the above-mentioned implant macrogeometric features and surgical technique [89,90,91]. Different levels of friction and bone-to-implant interlocking can be achieved, resulting in either high- or low- primary stability depending on factors such as the implant thread design interaction with the surgical instrumentation [92,99,100]. The continuous search for devices that can support atemporal loading has led to the development of new protocols that combine different bone healing pathways to ensure satisfactory primary stability and accelerated secondary stability [89,90,91,92,95,99,100,101,102,103,104,105,106]. In this context, implant macrogeometries with large thread designs and potential alterations in osteotomy dimensions have demonstrated hybrid healing pathways. Thus, primary stability is immediately achieved through the thread tip and then progressively improved by rapidly forming intramembranous-like woven bone [92,95,99,100,101,102,103,104,105]. While a lower extension of interfacial bone remodeling occurs at sites where the implant threads engage the bone (specifically for primary stability), the formation of woven bone simultaneously occurs in the healing chambers, thereby compensating for any stability decrease and speeding up the establishment of secondary stability [92,95,99,100,101,102,103,104,105]. This hybrid healing approach offers a potential alternative for implant treatments, which may lead to devices with long-term stability [89,90,91].

**Figure 4:**
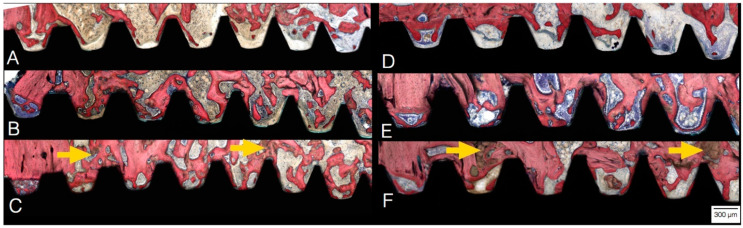
Histological micrographs of implants at 3 (**A**–**C**) and 6 weeks (**D**–**F**), respectively, where (**A**,**D**) represent conventional (subtractive/regular) drilling, (**B**,**E**) represent osseodensification with clockwise drilling, and (**C**,**F**) represent osseodensification with counterclockwise drilling. Orange arrows highlight the autologous bone chips compacted between the walls of the anatomy and implant surface as a result of the osseodensification drilling process, thereby acting as nucleating sites for newly forming bone. Reprinted with permission from the work by Oliveira et al. [120]. Copyright 2018. Published by Elsevier B.V.

Surgical instrumentation techniques for osseointegration in low-density bone have been continually evolving to maximize stability and to promote successful implant fixation. Conventional methods utilize subtractive drilling, where bone is “excavated” and removed to position the metallic device [107,108,109,110,111,112,113,114,115]. However, preserving bone bulk during osteotomy is crucial for stable implant placement [89,90,91,116,117]. Based on such premises, the osseodensification technique revolutionizes bone preparation by using an additive multi-stepped drilling concept with specially designed burs that densify the surrounding osseous environment instead of drilling bone out [118,119,120,121,122,123,124]. This method creates a layer of autologous bone graft with improved density and is beneficial in compromised bone conditions (Figure 4) [119,120,123,124,125]. Hence, osseodensification preserves bone fragments and enhances density through viscoelastic and plastic deformation [126]. The densified bone increases primary stability by maximizing the engagement with the implant [119,120,123,124]. Additionally, incorporating host bone chips, or autografts, into the bone–implant interface can accelerate new bone growth and stimulate peri-implant osteogenesis [119,120,123,124,125].

**Figure 5:**
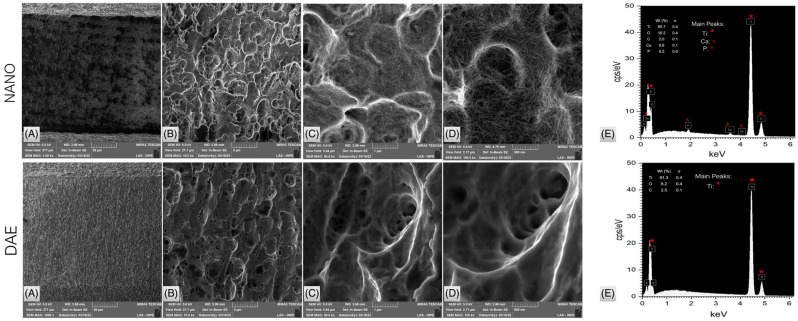
Scanning electron micrographs at (**A**) 100×, (**B**) 1000×, (**C**) 5000×, and (**D**) 10,000× and (**E**) energy dispersive X-ray spectroscopy (EDS) of the CaP-coated implants (NANO—top row), and dual acid-etched (DAE—bottom row) implants. EDS depicts the presence of Ca and P on the surface of the NANO implants, as seen by the prominent peaks in the spectra. Reprinted with permission from the work by Bergamo et al. [160]. Copyright 2024 Wiley Periodicals LLC.

Another method to accelerate osseointegration in conjunction with implant macrogeometry and surgical techniques is the use of an implant system with engineered micro- and nano-topographical features. From an engineering perspective, numerous topographical and chemical modifications to implants have been explored to expedite early bone healing around implants, especially in challenging scenarios such as low-quality bone or compromised systemic conditions [89,90,91]. Complex surfaces have shown to maximize osseoconduction by increasing the available surface area for protein adsorption and modulating the host response [118,120,127,128,129,130,131]. Recent developments have focused on nanoscale chemical modifications of implant surfaces and their impact on osteogenic potential. Nano topographical modifications were reported to improve osseointegration by altering both the texture and chemical properties, such as wettability [109,132,133,134,135,136,137,138,139,140,141,142,143,144,145,146,147,148,149,150,151,152,153,154,155]. Based on the fact that natural tissues are composed of biomolecular structures that vary in size from nano- to micro- and macro-scales, a recent surface modification strategy has been employed to apply hierarchical topographical structures that contribute to an accelerated and higher degree of osseointegration. This strategy aims to increase biomolecular interactions, leading to improved protein adsorption, osseoconduction, and osseoinduction [90,91,156,157]. Recently, nanostructured calcium phosphate (CaP) coatings on implant surfaces (Figure 5) have shown promising results in accelerating osseointegration. This has also been reported to have enhanced the mechanical properties of bone relative to conventional micro- and nano-topography surface modifications, potentially due to the osteoconductive properties of CaP, mimicking the structure and composition of the surrounding bone [157,158,159].

Improvements in dental implant coatings are the most recent clinical advancement in surface modifications [161]. Different coating methods that involve bioactive agents can be classified into biocompatible ceramics, bioactive proteins, ions, and polymers. Potential bioactive agents that have been investigated include growth factors, bone morphogenetic proteins, the RGD peptide, type I collagen, fluoride, and/or chitosan, to name a few [162]. In one study, sandblasted and acid-etched implant screws integrated with biomimetic surfaces coated with covalently immobilized collagen type I were utilized in rabbit articular femoral knee-joints, which resulted in the collagen positively influencing cell migration, attachment, proliferation, and differentiation [163]. Mimicking biology through implant modification (nanoscale alterations) by applying bioactive coatings might significantly improve the biological response as well. For example, platelet-rich fibrin (PRF) has been used in preclinical studies as a coating on polymeric scaffolds, demonstrating improved bone regeneration in qualitative and quantitative evaluations in vivo [164,165].

**Table 1:** Summary of main findings of biomimetic applications in the oral and maxillofacial setting.

Specific Field	Material Composition	Material Type	Study Design	Biological Properties	Clinical Application	Main Findings	Reference
Restorative Dentistry and Prosthodontics	Fluorapatite–gelatin composites (gelatin matrices)	Hydrogel	In vitro	Mimic formation on a lower level of complexity compared with teeth	Biomimetic Biomineralization for the formation of calcified tissue	The composite demonstrated a relation of the results with calcified tissue	[7]
	Gelatin gels loaded with calcium and phosphate	Hydrogel and bioceramic	In vitro	Fluoride ions were found, and mineralization of enamel-like layers was observed in enamel and dentin samples	Biomimetic mineralization	The composition using biomimetic agents resulted in an increase in the crystallinity and mineral content in enamel and dentin owing to fluoride, calcium, and phosphate penetration	[8]
	Ceramic adhesive restoration	Bioceramic	In vivo	Tissue preservation and adhesion	Ceramic adhesive restoration in the anterior area demonstrated the possibility of replacing a previous deficient crowns and devitalized teeth	Conservation of the biological, esthetic, biomechanical, and functional properties of enamel and dentin	[10]
	Calcium–silicate hybrid	Bioceramic	In vitro	Stimulate the formation of new apatite-containing tissue	Biomimetic remineralization of apatite-depleted dentin surfaces	Prevented the demineralization of hypo mineralized/carious dentin	[11]
	Agarose gel loaded with calcium phosphate	Hydrogel and Bioceramic	In vitro	Molecular mechanics of organic-matrix-mediated biomineralization of dentin surfaces	Dentin remineralization and a new method to treat dentin hypersensitivity and dental caries	Dentinal tubules were occluded, and hydroxyapatite crystals covered the dentinal surface	[12]
Endodontics	Calcium hydroxide pastes	Bioceramic	In vivo	Bactericidal effect conferred by the pH of the environment	Intracanal medicament for bactericidal effect	The paste can influence the pH, showing that alkalinity is an important factor	[64]
	Bioactive nanofibrous scaffolds	Polymer	In vitro	Antibiotic-containing scaffolds	Antimicrobial drug delivery system for regenerative endodontics to disinfect necrotic immature permanent teeth	The polymer-based antibiotic-containing electrospun scaffolds can provide a biologically safe antimicrobial drug delivery system for the regenerative endodontic field	[68]
	Electrospun polymer scaffolds	Polymer	In vitro	Antibiotic-containing scaffolds	Nanofibrous scaffolds can be used as an alternative for intracanal disinfection prior to regenerative endodontics	The antibiotic-containing nanofibrous scaffold demonstrated a capacity to be used against *Porphyronmonas gingivalis* infection into dentin biofilm	[69]
Periodontics	Electrospun nano-apatite composite membrane	Polymer	In vitro	The application of bone-like ceramics into the membranes can mimic the mineral crystals in the natural tissue and increase cell adhesion	The biodegradable membrane system can be used for guided tissue or bone regeneration	Electrospun membrane incorporating nano-apatite was strong, enhanced bioactivity and supported osteoblast-like cell proliferation and differentiation	[78]
	Electrospun membrane	Polymer	In vitro	The novel functionally graded membrane was developed with layers of nano-hydroxyapatite and metronidazole for optimizing periodontal regeneration	The periodontal membrane demonstrated osteoconductive behavior provided by nano-sized hydroxyapatite particles and metronidazole against periodontal pathogens	Incorporation of nano-apatite enhanced osteoconductive behavior and combatted periodontal pathogens	[79]
Implant Dentistry	Titanium–zirconium alloy	Metal and ceramic	In vitro	The mechanical properties of dental implants can play an important role during osseointegration	The lower elastic modulus and higher hardness of titanium–zirconium make this material stronger and more suitable for high-load-bearing dental implants	The addition of zirconium in titanium implants increases the strength property, which can be beneficial for high-load-bearing areas and lowers the elastic modulus which reduces the stress-shielding effect and eventually leads to implant failure	[84]
	Zirconium oxide implants and PEEK (polyether–ether–ketone) restorations	Ceramic	In vivo	Great biocompatibility, biostability, and mechanical properties	PEEK restorations can be applied with zirconia implants	PEEK restoration is a valid alternative when used with zirconium implants, demonstrating greater effect on the elastic modulus, which can absorb occlusal forces and wear like a natural tooth	[86]
	Nanocrystals of hydroxyapatite into titanium	Metal	In vivo	The nanostructure of dental implants improves osseointegration through biomimicry of the host structure	Improved osseointegration of dental implants	The capacity of nano-hydroxyapatite to strengthen bone quality could be observed	[145]
	PEEK (polyether–ether–ketone) implants	Thermoplastic	In vivo and in vitro	Cellular osteogenic differentiation and increased implant osseointegration for porous PEEK samples when compared with smooth PEEK and plasma-sprayed titanium on PEEK	Osteointegration of dental implants using PEEK material, bone ingrowth volume, and fixation strength	PEEK implant topography has a central role in implant osseointegration	[87]
	Titanium coated with collagen type I	Metal	In vivo	Collagen type I on the implant surface is efficient for accelerating early osseointegration and improving the bioactivity of the titanium implant surface	Osseointegration with dental implants coated with collagen type I may not increase the amount of bone in contact with the implant	Mineralization of bone around dental implants when coated with collagen type I	[163]

## 3. Biomimetics in Craniofacial Reconstruction

Due to its prominent position, the craniofacial region is susceptible to trauma that may result in defects requiring surgical intervention and reconstruction. Autografts, allografts, xenografts, and alloplasts are distinctly sourced grafts used in the repair and regeneration of craniofacial defects [166]. Autografts are clinically and historically considered the gold standard for craniofacial reconstruction because, in addition to their immunologically inert properties, they possess osteogenic and osteoconductive potential [166]. However, there are several disadvantages to autogenous bone grafting, such as donor site morbidity and associated complications.

Current alternatives for autogenous bone regeneration range from allogenous, xenogenous, and alloplastic bone grafts to synthetic scaffolds, which can be associated with different cell therapies and growth factors [167]. A study by Martín-Piedra et al. attempted to develop a highly biometric multilayered palate substitute consisting of bone and oral mucosa tissue [168]. Their study was the first of its kind to fabricate a multilayered model of a rabbit hard palate through a combination of tissue engineering and nano-structuration. The bioengineered palate bone substitute was created using rabbit adipose tissue mesenchymal stem cells that were treated with an osteogenic differentiation conditioning medium for 21 days to induce osteogenic differentiation, and the oral mucosa substitute was created using an oral mucosa stromal substitute treated with fibroblasts and subcultured with rabbit keratinocytes. A multilayered palate substitute was then fabricated via nano-construction. Bioengineered palates were then grafted autologously in each animal after a full thickness 4 mm defect was created on the right side of the palate. Histological analysis after 12 weeks showed that the bioengineered palate was able to integrate with the surrounding tissues in the animals and undergo a process of tissue differentiation. However, the expression of specific components, such as cytokeratin, proteoglycans, elastic and collagen fibers, mineralization deposits, and osteocalcin, remained significantly lower in the bioengineered palate when compared with the control [168].

On the other hand, scaffolds may also be designed using bioceramics, which are a subclass of ceramics that possess biocompatible, bioinert, bioactive, or bioresorbable properties [169,170,171,172,173,174]. CaP-based materials have recently garnered attention because of their excellent biocompatibility and strength in in vitro and in vivo models [175,176]. Hydroxyapatite (HA) and β-tricalcium phosphate (β-TCP) are two of the most widely researched CaP-based ceramics [177]. There is considerable heterogeneity in the types of HA being examined, as it appears in both natural and synthetic forms with varying physical microstructures, crystal sizes, and porosities [178]. The microstructure, encompassing the proportion of HA and its morphology and dimensions, significantly influences the osteoconductivity and biocompatibility. Biomimetic scaffolds utilizing HA in conjunction with chitosan have been demonstrated in the literature. More specifically, chitosan crosslinked with genipin (CTS-GP), and mineralized CTS-GP scaffolds containing hydroxyapatite (CTS-HA-GP) have been recently investigated [179]. Alkaline phosphatase activity, which is the protein associated with bone formation, on the CTS-HA-GP scaffold was approximately two times higher than that on the CTS-GP scaffold. Enhanced bone regeneration was observed with the use of the CTS-HA-GP scaffold relative to the CS-GP group. Additionally, pre-seeding with murine mesenchymal stem cells promoted increased bone healing [179]. Moreover, there is evidence that incorporating dopamine may promote the mechanical strength of hydroxyapatite-based scaffolds and further improve their biomimetic properties. Lee et al. compared the osteogenic effects of polydopamine-laced hydroxyapatite–collagen–calcium–silicate (HCCS-PDA) with hydroxyapatite–collagen–calcium–silicate (HCCS)-coated plates [180]. Both types of plates, with or without rat mesenchymal stem cells (rMSCs), were used in a rodent model. The study found that after twelve weeks, the HCCS-PDA group seeded with rMSC aggregates led to the formation of bony tissue throughout the defect, whereas the HCCS group seeded with rMSC aggregates did not evidence any bridging at the defect site [180].

**Figure 6:**
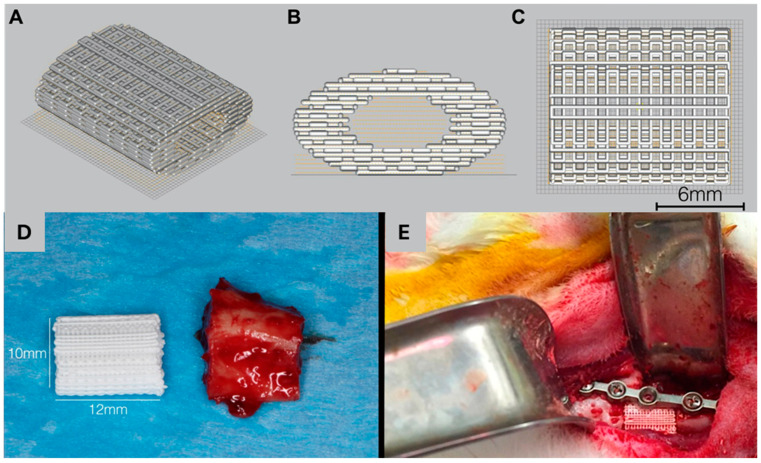
CAD of a cylindrical mandibular scaffold showing a concentric inner cylindrical cut-out (to mimic the lumen) in (**A**) isometric (**B**) front, (**C**) top views. (**D**) A surgically resected critically sized full-thickness mandibular segment shown as a side-by-side comparison to the 3D-printed β-TCP scaffold, and (**E**) surgical placement of the 3D-printed scaffold and stabilization of the scaffold within the critically sized defect site with surgical hardware. Reprinted with permission from the work by Lopez et al. [189]. Copyright 2017 Elsevier Inc.

The introduction of new osseoconductive biomaterials and methods of fabricating customized scaffolds, including computer-aided design (CAD)/computer-aided manufacturing (CAM) and 3D-printing techniques, have produced innovations associated with maxillofacial reconstruction [181]. One such ceramic, namely β-TCP, has garnered considerable interest as a viable substitute for autologous bone grafts. This is primarily attributable to its substantial capacity for reabsorption and replacement by natural bone, a unique characteristic relative to other commonly used ceramic materials [182]. β-TCP is also osteoinductive, facilitating osteogenesis at defect sites [183,184]. The combination of these properties and its resemblance to the mineral component of bone renders β-TCP a possible substitute for bone grafts. HA and β-TCP, although possessing analogous chemical compositions (calcium-to-phosphate molar ratios of 1.67 and 1.5 respectively), exhibit different resorption characteristics [183]. The resorption rate of TCP biomaterials is influenced by their morphology, chemical content, and structural features such as macro- and micropores [183]. This includes both cell-mediated resorption and chemical dissolution. In contrast, the lower degradation rate of HA has primarily been ascribed to a cellular phenomenon [183]. For instance, in a pre-clinical study, β-TCP scaffolds exhibited significantly greater resorption (~77%) relative to HA (~61%) after a 6-month period [185]. Other clinical investigations have asserted that β-TCP exhibits comparable rates of healing, clinical success, patient improvements, and radiographic results relative to autografts, alongside diminished therapeutic failures, pain, and/or complications [186]. Studies have also shown that the faster absorption kinetics of β-TCP (relative to HA) may be a favorable property for preventing long-term risks of exposure and infection [187,188]. β-TCP colloidal gels have also been developed to enable the 3D printing of scaffolds for critical-sized defects (Figure 6), with promising outcomes for potential future clinical trials, especially with the use of bioactive molecules and growth factors [166,188].

Although growth factors are normally secreted in the wound healing process, studies have investigated the effects of adding osteoprogenitor cells, such as stem cells, and growth factors to further promote the regenerative capacity of reconstructed bone and the surrounding tissue. The therapeutic impact of stem cells in tissue engineering, often in conjunction with scaffolds, has traditionally been achieved through the transplantation of exogenous cells into the host tissue during regeneration. However, recent studies suggest that paracrine signaling may also be a key mechanism driving the regenerative effects of these cells [190,191]. A stem cell’s secretome, which includes micro vesicles and exosomes [191], has been found to play an essential role in cell-to-cell communication, triggering a cascade of events that can influence bone metabolism and promote regeneration [192,193,194,195]. The secretome contains a variety of factors with anti-inflammatory, angiogenic, and immunomodulatory properties [191]; however, the identification of the ideal secretome profile in the regeneration process has yet to be addressed.

Regarding growth factors, the use of platelet concentrates (platelet-rich plasma (PRP) platelet-rich fibrin (PRF)), vascular endothelial growth factor (VEGF), fibroblast growth factor (FGF), bone morphogenetic proteins, and platelet-derived growth factor (PDGF) has been investigated in tissue engineering therapies [167,196,197,198,199,200,201,202,203]. One of the most potent growth factor in tissue engineering is bone morphogenetic protein [204]. This biomolecule is an osteoinductive protein that attracts osteoprogenitor cells, stimulating their growth and differentiation into mature osteoblasts while also increasing VEGF levels to enhance angiogenesis [204,205]. A study by Lee et al. investigated composites that may enhance in vitro osteogenic behaviors and accelerate in vivo bone growth [206]. In the aforementioned study, borosilicated Col/β-TCP composites (Col/Si-β-TCP) were created using a silica-forming peptide derived from a brown alga, which were then used to investigate the potential applicability of these composites as BMP-2-delivering bone-graft substitutes. Twelve male Sprague–Dawley rats were operated on to create a critical-size defect using an 8 mm diameter trephine. The defects were then replaced with either a Col/β-TCP block, Col/β-TCP@BMP-2 block, or Col/Si-β-TCP@BMP-2 block. A comparison across groups showed that rats in the Col/Si-β-TCP@BMP-2 group had the fastest bone healing rates after four weeks. This study showed how the biosilicification of β-TCP bone grafts can provide robust and sustained BMP-2 delivery, thereby enhancing in vivo bone regeneration [206]. Further highlighting the role of BMP-2 in effective bone regeneration is a study by Baskin et al., where the role of a nanophase bone substitute (NBS), a biomimetic material synthesized of type I collagen arranged in a D-staggered array, as an effective load-bearing bone substitute was investigated [207]. Four NBS renditions were implanted with and without BMP-2 in a rodent mandible-body critical-size defect. After nine to twelve weeks, the addition of BMP-2 to non-crosslinked samples led to an osseointegrated implant. The study also investigated the effect of crosslinking and high-pressure densification of the nanophase bone substitute and found that all crosslinked NBS implants formed a fibrous non-union with the surrounding mandible, and hyper-densification had little effect on cellular behavior. Together, this study showed that BMP-2, in the presence of non-crosslinked NBS grafts, permitted the osteointegration of implants [207].

This is in comparison to another study by DeCesare et al., who showed that recombinant human bone morphogenetic protein-2 (rhBMP-2) delivered on an absorbable collagen sponge in a rabbit model with a parietal defect resulted in undesirable skeletal changes [208]. Treatment with rhBMP-2 resulted in fusion of the coronal sutures bilaterally and variable fusion of the sagittal suture. This ultimately resulted in a significantly reduced maxillofacial length by 42 days and a 12% decrease in intracranial volume by 84 days [208]. Further highlighting safety concerns with the use of rhBMP-2 in the pediatric model was a study by Lopez et al. [200]. There is also evidence that higher concentrations of rhBMP-2 in scaffolds accelerated premature suture fusion. Liu et al. studied thirty 6-week-old rabbits treated with 0 (control), 0.1 mg/mL, or 0.4 mg/mL of rhBMP-2 delivered on an absorbable collagen sponge placed over the midsagittal suture as part of a midsagittal sutural expansion and found that rabbits treated with either 0.1 mg/mL or 0.4 mg/mL rhBMP-2 experienced premature suture fusion that significantly prohibited sutural separation between days 10 and 30 [209]. Higher concentration of rhBMP-2 accelerated bone growth in the first ten days of the experiment but, ultimately, led to premature suture fusion.

BMP-2 is approved by the FDA for use in craniofacial reconstructions, with recent reviews reporting successful clinical outcomes [203,204]. However, the effectiveness of tissue engineering therapies is directly related to the concentration of the biomolecule [203,204]. Some other side effects that have been reported are in sites where BMP has been used, such as increased inflammation compared with autogenous bone grafts, the formation of bone in unintended locations, and the production of antibodies against growth factors [203,210]. Therefore, future research should focus on developing more advanced carriers or biomolecules with controlled release properties that can lead to a more predictable pattern of bone formation [198].

**Figure 7:**
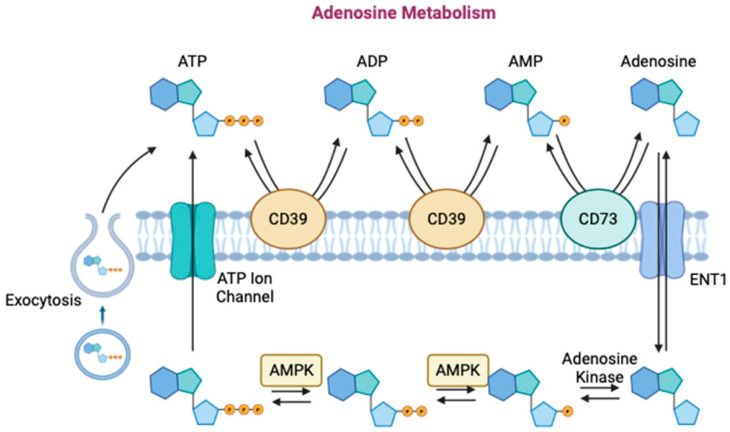
Schematic illustration depicting the extracellular concentration of adenosine through the effect of enzymes and adenosine metabolism. Abbreviations: ATP: adenosine triphosphate; ADP: adenosine diphosphate; AMP: adenosine monophosphate; CD39: ectonucleoside triphosphate diphosphohydrolase-1; CD73: 5′-nucleotidase, also known as ecto-5′-nucleotidase; ENT-1: type 1 equilibrative nucleoside transporter; AMPK: adenosine monophosphate-activated protein kinase. Reprinted from the work by Ehlen et al. [225] under the terms of the Creative Commons Attribution (CC BY) license (https://creativecommons.org/licenses/by/4.0/). Accessed on 7 August 2024.

In this context, activators of purinergic receptors (i.e., the adenosine receptor) that induce bone formation and mitigate or nullify adverse effects, such as those associated with BMPs, are under investigation (Figure 7) [199,211,212,213,214,215,216]. One such drug, dipyridamole (DIPY), acts as an indirect agonist of the adenosine A_2A_ receptor [199,200,201,202,217]. DIPY works by blocking adenosine reuptake into the cell via the type 1 equilibrative nucleoside transporter (ENT1), leading to extracellular adenosine accumulation [218,219,220]. The effectiveness of DIPY in improving hard-tissue regeneration has been successfully shown in multiple preclinical studies [199,200,201,202,217]. Bioceramic scaffolds, when combined with DIPY, facilitated localized drug delivery to promote bone regeneration while simultaneously avoiding unintended systemic effects [221,222,223]. This has been observed even at increased doses greater than the concentration required for bone formation [200,224] and during long healing timepoints, when scaffolds have fully resorbed and the effects of DIPY have diminished [176].

While BMP has been linked with unintended systemic side-effects such as premature suture fusion, DIPY has been shown to facilitate localized drug delivery when loaded on 3D ceramic scaffolds [166]. For example, a study compared DIPY with rhBMP-2 in alveolar cleft defects [200]. Unilateral alveolar resections adjacent to growing sutures were created in skeletally immature New Zealand white rabbits and treated with either DIPY- or rhBMP-2 loaded 3D-printed bioceramic scaffolds. While no changes in suture biology were observed on follow-up for rabbits treated with DIPY, early signs of suture fusion were evident in rabbits treated with rhBMP-2 [200]. The regenerative capacity of β-TCP scaffolds, with and without the incorporation of bioactive molecules, has been extensively investigated in preclinical translational animal models using critical-size bone defects [176,188,189,199,201,202,226,227]. Bioactive ceramic scaffolds composed of 100% β-TCP in a methodical design with various length scales and dimensions (2D and 3D) in a rabbit model for segmental mandible defects demonstrated newly formed bone as early as 8 weeks, with evidence of approximately 20–40% bone regeneration, and up to 80% bone regeneration at 24 weeks [176,189,202]. Concomitant with bone formation, progressive scaffold resorption over a 24-week healing period could be observed [176]. The longest in vivo study using ceramic scaffolds coated with DIPY for the regeneration of alveolar defects in a rabbit model indicated that bioactive ceramic scaffolds seamlessly fit, filled, and bridged the defects with newly formed bone (50% of the original bone volume), and that scaffold degradation progressively occurred along with further bone formation and remodeling [188]. Similar principles were also applied and successfully demonstrated in a larger, more clinically relevant model [199].

In cases involving cleft palate reconstruction in pediatric patients [200,224,228], the ideal bone replacement would effectively fit and fill the defect site and restore structure and function while preserving bone growth. As such, a study was conducted where the defects were treated with 3D-printed bioactive ceramic scaffolds coated with type I collagen versus scaffolds augmented with either 10^2^, 10^3^, or 10^4^ µM DIPY [200]. Dose-dependent bone regeneration with no evidence of maxillary suture fusion was observed, with 120%, 130%, and 100% higher bone formation for 100 µM, 1000 µM, and 10,000 µM DIPY relative to collagen, respectively [200]. In addition, the regenerated bone presented similar characteristics to positive-control animals treated with autologous bone grafts, with no signs of asymmetry, ectopic bone growth, or morbidity. In a similar study, no significant difference was noted in bone growth and resorption kinetics between the alveolar defects repaired with 3D-printed DIPY scaffolds and an autologous bone graft at 24 weeks [228]. Also, the effectiveness of 3D-printed β-TCP scaffolds in regenerating pediatric maxillofacial defects has been reported in a larger pre-clinical translational animal model using 6-week-old Göttingen minipigs, which is an excellent animal model due to its similarities to human bone morphology, regeneration, and wound healing, as mentioned above [229,230].

Nonetheless, the available animal models are deficient in predicting clinical performance in several areas where the combined deficits of hard and soft tissue might compromise the healing process [231]. Furthermore, the defects used in all the aforementioned animal models involve grafting into a biological environment characterized by a clean, freshly cut vascular bone and a relatively untraumatized and well-vascularized soft-tissue envelope [231]. Future pre-clinical studies should be implemented with a better representation of the complex clinical environment, such as in compromised wound healing models.

### 3.1. Engineering Strategies for Biomimetic Micro- and Nano-Architectures

Bone exhibits a hierarchical porosity, ranging from a few microns to several hundred microns. As such, in the application of biomaterials, it is crucial to consider factors that influence the formation of new bone. The effective regeneration of bone by tissue engineering depends on the ability to mimic the natural environment to enhance cellular adhesion and differentiation. The fabrication of biomimetic micro- and nano-architectures is, therefore, essential to simulate the intricate structures of native craniofacial tissues [232]. Craniofacial bone scaffolds should ideally possess mechanical properties comparable to those of native bone. Stiffer scaffolds may induce stress shielding, leading to bone resorption over time. However, it is generally difficult to synthesize porous scaffolds that possess adequate mechanical strength for load-bearing applications, as the high porosity necessary to promote vascularization and cellular infiltration during tissue regeneration compromises strength/stiffness. Nonetheless, microarchitectural designs and surface topography are essential elements in the functionalization of scaffolds. Surface topography is essential for optimizing scaffold function by augmenting cell adhesion and proliferation via mechanical signals transmitted to cells [232]. Alongside particular biochemical signals, these physical cues serve as broader regulators of cellular activity and function [233]. Enhancing the surface physicochemical characteristics of scaffolds facilitates improved cell adhesion and initiates the regenerative process. Numerous procedures exist for enhancing topography; common examples include acid etching and plasma treatment [234,235,236]. Conversely, microarchitectural characteristics that affect performance include the pore size and morphology, porosity, surface area to volume ratio, and mechanical strength.

Several engineering strategies have been developed to achieve porous constructs, including electrospinning, 3D bioprinting, self-assembly, lithography, and layer-by-layer assembly. Electrospinning produces nanofibers that mimic the extracellular matrix (ECM) structure and has been used to promote bone and periodontal regeneration in craniofacial application [237]. Three dimensional (3D) bioprinting allows precise control over the scaffold architecture and cell placement, enabling the creation of patient-specific implants and tissue constructs [238]. Self-assembly mimics the natural processes of tissue formation, with peptide-based self-assembling nanofibers showing promise in bone regeneration [239]. Lithography enables the creation of precise micro- and nano-patterns, with nanolithography being used to develop surfaces that guide cell behavior for improved osseointegration [240]. Layer-by-layer assembly allows fine control over the material composition and properties and has been applied to create biomimetic, coated bone scaffolds [241]. These strategies also have other applications, such as in periodontal regeneration using multilayered scaffolds that mimic the complex structure of the periodontium, dental pulp regeneration with nanofibrous scaffolds that support odontoblast differentiation, and enamel regeneration through biomimetic mineralization techniques that replicate enamel’s hierarchical structure [238].

### 3.2. Functional Outcomes and Considerations

The reconstruction of the craniofacial region can be executed by various surgical procedures and facilitated by accurate/customized cutting guides, fixation devices, and scaffolds. These methods and tools may be utilized separately or in combination to treat a specific craniofacial defect. A prevalent theme, however, is the necessity for individualized therapies to attain both an esthetic appearance and complete functional restoration. Consequently, 3D-printing methodologies capable of producing intricate craniofacial geometries are ideally suited for meeting these specific requirements. In previous studies, DIPY-augmented β-TCP scaffolds have been investigated in critical-sized craniofacial defects. In one noteworthy example, Shen et al. reported accelerated degradation of β-TCP scaffolds to approximately 55% in calvarial defects and up to 90% in alveolar defects within a 12-month period [188]. β-TCP scaffolds were replaced with regenerated, vascularized bone with similar mechanical and histologic properties to those of native bone [188]. When evaluated in skeletally immature animal models, bone regeneration using DIPY-coated bioactive ceramic scaffolds evidenced suture patency [188]. On a related note, facial development post-surgical-implantation of scaffolds could result in asymmetric bone growth. However, a previous study by Wang et al. showed a lack of facial asymmetry with the use of biomimetic tissue engineering strategies, specifically porous 3D-printed bioactive scaffolds, through 3D morphometric analysis and cephalometry [228].

## 4. Future Directions

With continuous advancements in various fields of science and technology, biomimetics has emerged as a promising area of research, particularly in dentistry and oral and craniofacial reconstruction. By imitating the biological structures, processes, and functions found in living tissue, biomimetics has the potential to revolutionize these fields, offering more efficient solutions for better patient outcomes. Biomimetics in dentistry aims to develop materials and techniques that closely mimic natural teeth, allowing for better functional and esthetic outcomes. Traditional restorative materials, such as polymers and ceramics, do not replicate the dynamic properties of natural teeth. Biomimetic dental materials with a gradient of properties that reproduce those of natural teeth should be developed, especially with the incorporation of new technologies like 3D printing that allows for the fabrication of layered systems. Antimicrobial properties, which reduce the risk of secondary infections and promote better oral health, would also be important aspects to incorporate into the dental materials to compensate for the hostile oral environment. Additionally, future research may focus on novel biomaterials that not only possess the ability to repair and regenerate tooth structures such as the enamel and dentin, but also the pulp, periodontal ligament, bone, and other surrounding tissues.

The replacement of missing teeth with dental implants has also revolutionized restorative dentistry by providing long-lasting prosthetic solutions. However, achieving optimal osseointegration and minimizing implant failure rates remain ongoing concerns. Future research could improve the design of implant surfaces by enhancing the adhesion and proliferation of osteoprogenitor cells, leading to faster and more effective/optimized integration with the surrounding bone tissue. Additionally, biomolecules, which are already under development, can be widely incorporated into implant surgery protocols to improve the biological response. The application of tissue engineering, particularly the use of growth factors like BMPs, scaffold materials, and novel cell lines, is expected to play a pivotal role in optimizing bone regeneration and defect repair. As research progresses, these biomimetic strategies are likely to become central to achieving superior reconstructive results, moving beyond traditional methods to more sophisticated, biologically inspired solutions [1,242].

While the future of biomimetics in dentistry and craniofacial reconstruction appears promising, several challenges must be addressed. First, the translation of biomimetic research into clinical practice requires rigorous testing and validation. A 2019 systematic review indicated that patients who underwent bony reconstruction using 3D printing technology achieved surgical success, resulting in both immediate and long-term enhancements in their bone structure and/or clinical conditions [243]. Of note, a clinical trial by Sumida et al. demonstrated that custom-made titanium scaffolds for guided bone regeneration in the mandible possessed an optimal shape, improved surgical handling, and significantly reduced the intraoperative duration, thereby mitigating postoperative infections and mucosal rupture [244]. The precision of the manufacturing process resulted in enhanced practical and esthetic outcomes [244]. However, the long-term safety, durability, and efficacy of biomimetic materials and techniques must be thoroughly evaluated through numerous follow-up clinical studies. Additionally, developing cost-effective and scalable manufacturing processes for biomimetic materials is essential to ensure their widespread application.

## 5. Conclusions

Biomimetics in dentistry and craniofacial reconstruction presents an exciting frontier in scientific research and clinical practice. This field brings nature-inspired innovations to the world of dental materials, restorative techniques, and craniofacial reconstruction. By mimicking natural structures and processes, biomimetic approaches have the potential to provide stronger, more durable, and esthetically pleasing solutions for patients. With continued research and development, we can expect biomimetics to reshape the future of dental, oral, and craniofacial surgery, improving patient outcomes and revolutionizing these fields.

## Data Availability

No new data were created or analyzed in this study. Data sharing is not applicable to this article.
